# Mobile Apps to Support the Self-Management of Hypertension: Systematic Review of Effectiveness, Usability, and User Satisfaction

**DOI:** 10.2196/10723

**Published:** 2018-07-23

**Authors:** Tourkiah Alessa, Sarah Abdi, Mark S Hawley, Luc de Witte

**Affiliations:** ^1^ Centre for Assistive Technology and Connected Healthcare School of Health and Related Research University of Sheffield Sheffield United Kingdom

**Keywords:** mobile phone, mobile application, mobile app, self-management, hypertension, blood pressure

## Abstract

**Background:**

Hypertension is a chronic disease that is considered to be a public health problem and requires efforts by patients to manage themselves. The global growth in the use of mobile phones and tablets has been accompanied by the increased use of health apps. Many of these apps support the self-management of hypertension and, therefore, they have the potential benefits of lowering blood pressure. Despite this, there is currently a lack of evidence for their effectiveness, usability, and patient satisfaction with their use.

**Objective:**

A systematic review was conducted to assess the effectiveness of apps in lowering blood pressure, as well as their usability and patients’ satisfaction with their use.

**Methods:**

We conducted searches in the following databases: MEDLINE (OVID), EMBASE (OVID), PsycINFO (OVID), CINAHL, the Cochrane Central Register of Controlled Trials (CENTRAL, The Cochrane Library), IEEE Xplore ASSIAN, Google Scholar and the main Arabic databases Al Manhal, AskZad, and Mandumah. We looked for studies that used apps in the self-management of hypertension from 2008-2016. We also checked the reference lists of the review papers and all the primary studies for additional references.

**Results:**

A total of 21 studies with a total of 3112 participants were included in the review. Of the 14 studies that assessed the effectiveness of the apps in lowering blood pressure, 10 (71.4%) studies (6 RCTs and 4 nonrandomized studies) reported that using the apps led to significant decreases in blood pressure and seemed to be effective in the self-management of hypertension. Of these 10, only 2 (20%) RCTs and 3 (30%) nonrandomized studies had a low–moderate risk of bias. The results of this review are inconclusive regarding which combinations of functionalities would be most effective in lowering blood pressure because of variation in the studies’ quality, but the data suggest that apps incorporating more comprehensive functionalities are likely to be more effective. In all the studies that assessed the usability of the apps and users’ acceptance of them, all the apps seemed to be accepted and easy to use.

**Conclusions:**

Most of the studies reported that apps might be effective in lowering blood pressure and are accepted by users. However, these findings should be interpreted with caution, as most of the studies had a high risk of bias. More well-designed, large-scale studies are required to evaluate the real effect of using apps in lowering blood pressure and to identify the most effective functionality combinations for lowering blood pressure.

## Introduction

Hypertension, in which the blood pressure (BP) in the arteries is raised, is one of the most common chronic diseases in adults. Patients can be diagnosed with hypertension when their systolic blood pressure (SBP) and diastolic blood pressure (DBP) are above 140/90 mm Hg, respectively [1]. Hypertension has been recognized as a major risk factor for many diseases, such as renal failure, heart disease, and stroke [1]. Despite the effect of lowering BP on reducing the risk of renal and cardiovascular disease, most people with hypertension poorly control their BP [2]. Therefore, it is important to encourage patients’ involvement in controlling their BP.

Self-management is considered an important element of chronic care management [3]. Self-management demands an active role of patients in managing their symptoms, treatment, psychosocial and physical effects, and changing lifestyle [4-6]. Achieving an optimum level of self-management behavior is difficult and requires considerable effort from patients. Mobile health technology (mHealth), defined as the use of mobile devices to deliver health care [7], has the potential to facilitate and optimize patients’ self-management [8-11]. This can be performed by integrating health care with everyday life by delivering and collecting health information and services in a convenient, accessible, and interactive mode [12,13]. The use of the new generation of these mobile devices, including mobile phone and tablets, has increased rapidly in recent years, and it is estimated that by 2018 mobile phones will be used by one-third of the global population [14]. Mobile phones have become an important platform to deliver health to patients through health apps. The rapid growth in the use of these devices has been accompanied by a huge expansion in health and health-related behavior apps, and more than 100,000 of these are used by millions of people [14,15]. Many health apps are targeted to support people with hypertension in their self-management by offering self-monitoring activities, reminders, tailored information, and feedback [16,17].

To the best of our knowledge, despite the potential benefits of apps for people with hypertension and the increased use of these apps, a synthesis of studies on their effectiveness in this population has not been conducted. This systematic review will synthesize the existing evidence on the effectiveness of apps in lowering BP, as well as their usability and patients’ satisfaction with their use.

## Methods

A systematic review was conducted and reported per the PRISMA statement for systematic reviews [18,19].

### Eligibility Criteria

The inclusion criteria were dependent on PICOS [18] as described below:

#### Population

The population was people with hypertension (18 years of age and over) and health care professionals (HCPs) supporting people with hypertension in their self-management in any care setting, without limitations on the participants’ gender, age or socio-demographic characteristics. Studies about people with chronic illness including hypertension as one of their inclusion criteria were also included.

#### Intervention

The intervention was a mobile phone or a tablet app that collects data, provides feedback, connects with HCPs or informs about hypertension to support the self-management tasks of hypertension. These tasks include self-monitoring of BP and other biometrics, healthy eating and drinking, being physically active, maintaining a healthy weight, adhering to medication, and managing stress and coping [1]. The app should also enable interactions between the user and the device via a set of interfaces (eg, a visual user interface). Studies in which a health app was the only method of delivery or in which it was a component of a blended intervention were also included.

#### Comparator

The comparator was either usual care or any other control intervention. Articles with no comparison were also included.

#### Outcomes

The outcomes of studies that were considered are: levels of BP, SBP, and DBP, as well as usability, attitudes, and satisfaction with mobile apps.

#### Study Designs

The eligible study designs were all quantitative, qualitative, and mixed-method studies that explore the self-management of hypertension using apps. Pilot studies were included because they might enable us to understand the status of apps.

### Data Sources and Search Methods

The electronic databases EMBASE (OVID), MEDLINE (OVID), PsycINFO (OVID), the Cochrane Central Register of Controlled Trials (CENTRAL, The Cochrane Library), CINAH, ASSIAN, and IEEE Xplore were searched, as was Google Scholar. Hand searching through the reference lists of included studies and systematic reviews was also conducted to find more related studies. These databases were searched using the concepts of hypertension, mobile apps, telemonitoring, and self-management (see Multimedia Appendix 2 for the MEDLINE search strategy). The search strategy was limited to English research published from 2008, when the first app store was launched, [12] to June 25, 2017.

### Exclusion Criteria

Studies were excluded based on the criteria in Textbox 1. Conference abstracts, protocols, commentaries or editorials or studies not in English or Arabic were not included.

### Study Selection

Reference management software (Endnote) was utilized to collect results from databases, and to de-duplicate articles. Two reviewers (TA and SA) independently scanned titles against the eligibility criteria and in a second phase the abstracts of selected titles. Cohen kappa was calculated to determine the agreement between the reviewers for each step of selecting titles and abstracts.

**Exclusion criteria.**

1. They were not aimed at hypertension or studies focusing only on primary prevention of hypertension or hypertension during pregnancy.
2. They examined interventions accessed by a personal digital assistant, desktop computer, laptop, netbook
3. They examined interventions accessed by a mobile phone or traditional tablet that did not permit participants to download or use any app from the app store.
4. They solely used messaging including short message service (SMS) text messaging, multimedia messaging service (MMS), websites, calls, emails or Web-based apps.
5. A mobile device was used to transmit information provided by a blood pressure monitoring device to care providers or clinicians, but in which there was no interaction with the user.
6. They describe only the technological development of a mobile system.

Titles and in the second phase abstracts received 2 points if they met the criteria, zero if not and 1 point when there was doubt. If the sum of reviewer scores for a title was 2 or more, the study was included for the next phase. Otherwise, it was excluded. Two reviewers separately reviewed the full articles when the total scores for the abstract equaled 2 points or more. Any disagreements were resolved through a discussion with other researchers (LdW and MSH).

### Data Extraction and Quality Assessment

Two reviewers independently (TA and SA) extracted data and assessed the quality of the included studies. Any disagreement was resolved through a discussion with other researchers (LdW and MSH) until consensus was reached.

Data were extracted using a standardized form, which was piloted by the reviewers. The Cochrane Collaboration’s Risk of Bias Tool was utilized to assess randomized controlled trials (RCTs) [20]. Nonrandomized quantitative studies were evaluated using 3 tools provided by the US National Institute of Health (NIH), March 2014 version: 1 for observational studies, 1 for controlled studies, and 1 for pre-post studies without control group [21]. The Critical Appraisal Skills Programme (CASP) was utilized for the quality assessment of qualitative studies [22].

### Data Synthesis and Analysis

An overview of the basic characteristics of the studies, including the intervention, population, and outcome, was summarized in a table. Data were not combined because of differences in the designs of the studies. A narrative synthesis was conducted instead [18,23]. All research findings were classified according to review objectives.

## Results

### Summary of Search Results

**Figure 1:**
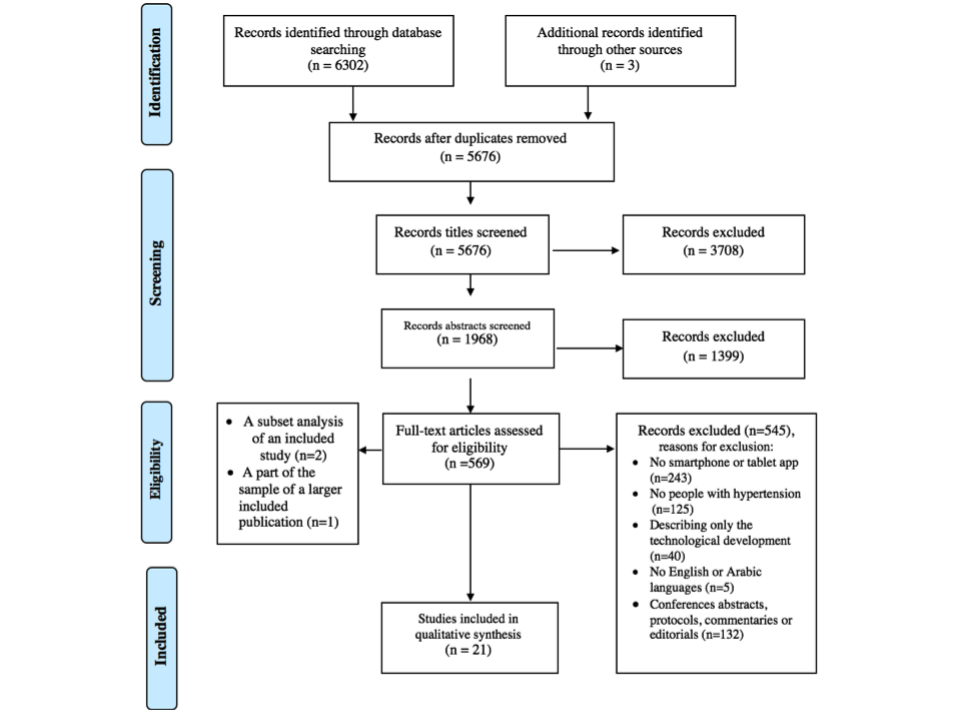
PRISMA flow diagram.

The review steps are summarized in Figure 1. Searching the electronic databases yielded a total of 6302 titles. After all duplicates were removed, 5676 records remained for title screening. Cohen kappa for agreement between the 2 reviewers was 0.72. Subsequently, the 2 reviewers (TA and SA) assessed the remaining 1968 abstracts; Cohen kappa for agreement between them in that step was 0.83. Of these, 569 went forward for full-text assessment, supplemented by 3 studies identified from reference tracking. A total of 548 papers were excluded at full-text screening, as they did not meet the criteria relating to the participants or interventions, or they were conference abstracts, editorials, or protocols. This led to a selection of 24 publications. Only 21of these were included in this review, as 2 publications were a subset analysis of a previous publication, and 1 publication was about a part of the sample of a larger study described in another publication.

### Study Characteristics

There were 21 studies included in this review. The publication year of the studies ranged from 2012 to 2017 (see Multimedia Appendix 1). Most studies (11/21, 52%) were conducted in the US [24-32] and Canada [33,34], while 7 (33.3%) were carried out in European countries, including France [35], Sweden [36-38], Spain [39,40] and Italy [41]. The remaining 3 (14.3%) studies were conducted in China [42,43] and South Korea [44].

Of the 21 studies, 9 (43%) were randomized controlled trials (RCTs) [24,25,27,28,30,33-35,42], 10 (48%) were nonrandomized studies [26,29,31,32,37,39-41,43,44], and 2 (10%) were qualitative studies [36,38].

Fourteen (14/21, 67%) studies reported on the apps’ effectiveness in controlling BP. Of these studies, 4 (27%) also assessed user satisfaction and experience with the apps [27,30,31,39]. The remaining 7 (33%) studies that did not report efficacy focused on user satisfaction with and attitudes towards the apps and their usability [26,32,36,38,40,43,44]. The study duration ranged from 1-12 months. The studies included a range of 19 to 1012 participants, with a total of 3112 participants.

Participants’ mean age ranged from 42.4 [27] to 69.5 [42] years of age. The population groups of the studies included individuals with hypertension [24-30,32,36-39,41-44], metabolic syndrome risk factors [34], obstructive sleep apnea with high cardiovascular risk [35], and overweight individuals [31]. Of the 21 included studies, 5 (24%) reported to having used behavioral theories, such as self-determination theory [24,26,27], motivational interviewing [30] and theory of planned behavior [43] to underpin and guide the intervention methods and the development of the technology. The other studies did not report using behavioral theories. However, an investigation of the apps’ functionalities identified recognizable elements of behavioral strategies.

All the included studies focused on supporting self-management of hypertension. Nine (43%) of the included studies were aimed to enhance self-management without involving clinicians to monitor patients remotely [29,33,35-38,42-44]. The other 11 (52%) studies mainly involved clinicians or other HCPs remotely monitoring patient data and health status [24-28,30-32,39-41], while the remaining study involved the researcher remotely monitoring patient data and alerting physicians if needed [34,36]. In these 11 (52%) studies involving HCPs, the HCPs provided feedback, including a medication plan or adjustments [24-27,39,41], regular online coaching consultation, [31] instructions [28,30], or communication with patients [40,42] (see Multimedia Appendix 1).

### Intervention Characteristics

In most studies, an app was supplemented with other interventions, such as a website [28,36-39,41], voice telephone messages [33], exercise prescription [34], a nasal mask and an auto-titrating machine [35], an electronic medication tray, email, SMS, or phone call [24-27], and education provided by a nurse [28]. The control group in the controlled studies had usual care. In some studies, this was combined with the recording of prescribed exercise [34] and the BP measurements [42] in a logbook or with the education provided by a nurse [28].

### Functionalities of the Apps

The 21 reviewed studies used 16 apps. Fourteen different apps were used in 14 studies [28-35,39-44], 1 app was used in 3 studies [36-38], and another app was used in the other 4 studies [24-27].

The main functions of the apps can be categorized into the strategies involved: self-monitoring capabilities, goal setting, the reminder and alert component (the use of prompts or cues), automatic feedback, educational information, communication with HCPs and stress management. All 16 apps incorporated at least one of these functions. Table 1 summarizes the characteristics of the apps and systems.

The 16 apps have some similar characteristics and functionalities. All the apps have self-monitoring capabilities for BP and other health data (medication adherence, physical activity, eating and drinking, weight, sleep, stress, symptoms, medication side effect, and self-reflection answers) [24-44]. This enables the user to track their BP and other health data over time in different formats, including graphical and/or tabular formats, and access the summary, raw data and/or analyzed results, the majority of which consisted of the BP, medication adherence, physical activity, eating and drinking, weight, and stress. The second most common functionality was a reminder and alert component that prompts self-monitoring by reminding patients about their medication time, BP measurements, hospital visits or personal goals, or the system alerts another person (eg, health professional) when a medication dose is missed or when the BP is higher than the normal level, a feature included in 13/16 (81%) apps [24-29,31-34,36-42,44].

**Table 1:** Intervention characteristics (identified by a check mark if they were met).

Study	Functionalities					
	Self-monitoring	Use of prompt/cues (reminder and alert)	Educational information	Communication with others	Automatic feedback	Stress management
Anglada-Martínez et al [39]	✔	✔	✔	✔	✔	—
Bengtsson et al [36]	✔	✔	—	—	✔	—
Bengtsson et al [37]	✔	✔	—	—	✔	—
Hallberg et al [38]	✔	✔	—	—	✔	—
Carrera et al [40]	✔	✔	✔	✔	✔	—
McGillicuddy et al [25]	✔	✔	—	—	✔	—
McGillicuddy et al [26]	✔	✔	—	—	✔	—
Davidson et al [24]	✔	✔	—	—	✔	—
McGillicuddy et al [27]	✔	✔	—	—	✔	—
Bloss et al [28]	✔	✔	✔	✔	✔	—
Patel et al [29]	✔	✔	✔	—	—	—
Or and Tao [42]	✔	✔	✔	—	✔	—
Logan et al [33]	✔	✔	—	—	✔	—
Petrella et al [34]	✔	✔	—	—	—	—
Albini et al [41]	✔	✔	✔	—	—	—
Mao et al [31]	✔	✔	✔	✔	—	—
Moore et al [30]	✔	—	—	✔	✔	—
Mendelson et al [35]	✔	—	✔	—	—	—
Kang et al [44]	✔	✔	✔	—	✔	—
Sun et al [43]	✔	—	—	—	—	—
Banerjee et al [32]	✔	✔	—	✔	✔	✔

Educational information [28,29,31,35,39-42,44] and automatic feedback [24-28,30,32,33,36-40,42,44] were the next most common features. Of 16 apps, 6 (38%) apps provided a tool for the users to communicate with their families and HCPs [28,30-32,39,40] and 1 app (6%) supported stress management [32]. Although setting goals is one of the most important techniques in the self-management of hypertension [45], most included studies reported that goals were set through negotiation and discussion between the patients and their HCPs without explicitly mentioning setting them in the app [24-27,29-31,34-38,42].

The most common comprehensive combination of strategies was self-monitoring, educational information, automatic feedback, reminders, and alerts. This combination was found in 5/16 (31%) apps [28,39,40,42,44], 3 of which also provided communication with HCPs [28,39,40], and patients families [28]. The second most frequently used combination was self-monitoring and prompt or cue, with the addition of either feedback [24-27,32,33,36-38] or educational information [29,31,41], with 2/7 (29%) apps providing communication with HCPs [31,32]. The remaining 4/16 (25%) apps only focused on self-monitoring [30,34,35,43] with either educational information [35], automatic feedback and communication with HCPs [30] or reminders or alerts [34] (see Table 2).

Regarding the automatic feedback feature, feedback was provided to participants using different approaches, either active feedback through self-care messages [33,40,44] and reinforcement messages [24-27], and passive feedback by representing data in different color codes to indicate whether measurement levels deviated from the normal range [28,32,40,42]. The communication with HCPs was through text messaging chats in the apps [30-32,39,40], with 1 of these 5 apps (20%) [31] adding consultations via video chats or calls in the app.

### Data Input Methods

Most apps (14/16, 88%) used self-monitoring of BP and supported other self-monitoring tasks [24-28,30-38,40-44], while 2 apps (13%) focused solely on self-monitoring of medication compliance [29,39]. In 50% (7/14) of the apps, the collected BP readings were transmitted automatically from BP monitoring devices to the app using wireless transmission. In 3 of these 7 apps (42.9%), Bluetooth was employed [24-27,33,34] while for the remaining 4 apps (57%) the transmission method was not described [28,30,35,42]. Manual entry of BP data was used in 50% of apps (7/14) [31,32,36-38,40,41,43,44], one of which (14%) also automatically transmitted data [31].

**Table 2:** Common combinations of app functionalities (N=16).

Common Combination	n (%)
Self-monitoring + automatic feedback + prompt or cue (reminders and alerts) + educational information	5 (31)
Self-monitoring + prompt or cue (reminders and alerts) + automatic feedback	4 (25)
Self-monitoring + prompt or cue (reminders and alerts) + educational information	3 (19)
Self-monitoring + communicate with health professional + automatic feedback	1 (6)
Self-monitoring + prompt or cue (reminder and alerts)	1 (6)
Self-monitoring + educational information	1 (6)
Self-monitoring	1 (6)

Blood glucose readings were also wirelessly transmitted in 3 of the 16 apps (19%) [28,34,42] and medication data was wirelessly transmitted in 2 of the apps (13%) [24-27,30]. There was no description of the technology used. Data was inputted manually in 3 other apps (3/16, 19%) using different formats, such as choosing an option or typing [29,39,44]. Other manually inputted data include: weight in 4 apps (25%) [31,32,34,41], number of steps walked in two apps (13%) [31,34], reflective answers representing users’ expectations toward their BP readings in one app [43], answers to questions about well-being, side effects, symptoms, and medication in another app [44], and other lifestyle aspects such as smoking, stress, and exercise in 2 apps [32,44].

### Quality Appraisal

All 9 RCT studies presented some degree of potential bias when assessed using the Cochrane Collaboration’s Risk of Bias Tool. Three of them were of low to moderate risk of bias (fair-good quality) because they met most of the criteria [33,35,42], while the remaining studies were considered to be of high risk of bias (poor quality) [24,25,27,28,30,34] (see Multimedia Appendix 4). Four of the 9 studies (44%) failed to report and apply random sequence generation [24,25,27,28]. Seven of the 9 studies (78%) presented a high risk of bias or information was not explicitly provided regarding the blinding of participants, personnel, or the outcome assessor [24,28,30,33-35,42]. Five (5/9, 56%) studies had a high risk of bias in other areas, such as small sample size [24,25,27,30,42].

One controlled study presented poor quality because of failure to apply blinding of the outcome assessor and sample size justification (see Multimedia Appendix 5). Most observational studies (4/7, 57%) were found to be of poor quality because of a high risk of bias or the lack of information concerning the sampling method and selection [32,39,43,44], and failure to clearly report the study aims, design, duration, and outcome measures [32,40], as well as high attrition rate [39,44]. The remaining 3 (43%) studies were of fair-good quality [26,31,37] (see Multimedia Appendix 6). One of the pre-post studies (1/2, 50%) presented poor quality because of selection and attrition bias [39] (see Multimedia Appendix 7). The two qualitative studies were deemed to be of low risk of bias as they met most of the CASP tool’s criteria. However, both seemed to fail to adequately report the saturation of data during data collection and the relationship between researcher and participants (see Multimedia Appendix 8).

### Blood Pressure

Fourteen studies (14/21, 67%) reported outcomes related to BP [24,25,27-31,33-35,37,39,41,42]. From these, 9 studies (64%) were RCTs [24,25,27,28,30,33,34,35,42], and 5 (36%) were nonrandomized studies [29,31,37,39,41]. Only 2 (14%) of them did not report the effect on DBP [25,31]. BP outcomes were presented as mean [25,27,29,39], mean change [24,28,30,33,37], or both [31,34,41,42] (see Multimedia Appendix 3).

As shown in Table 3, 6/9 (67%) studies demonstrated positive effects on BP [24,25,27,30,33,42], whereas 3/9 (33%) studies reported no positive impact on BP [28,34,35]. The 6 studies that demonstrated positive effects showed a significant decrease in SBP (*P*<.05). The decrease in the intervention arm ranged from 8.7 to 34.8 mm Hg [24,25,27,30,33,42]. Significant decreases in DBP were reported in 2/6 (33%) studies, ranging from 4.9 to 12 mm Hg [24,33]. Only 1 of the 6 studies (17%) [30] reported a nonsignificant trend toward greater decrease.

Three out of 9 studies (33%) were of fair-good quality. However, the remaining 6 studies (67%) were of poor quality (see Quality Appraisal section for an in-depth discussion of this). Of the 3 studies that were fair-good quality, only 2 (67%) were positive. Five of the studies (5/14, 36%) are nonrandomized [29,31,37,39,41]. Of these, 4 (80%) reported a significant decrease in BP [29,31,37,41]. This decline ranged from 5.7 to 10.5 mm Hg and from 4.9 to 6.2 mm Hg for SBP and DBP respectively (see Table 4). Three of the 5 (60%) nonrandomized were of good-fair quality and 2 (40%) of the studies were of poor quality (see Quality Appraisal section).

Of the 6 studies with low-moderate risk of bias, 1 (17%) reported no significant effect on BP [18]. Five studies, 2 of which were RCTs (40%) [33,42] that reported positive impacts on BP. Most of these studies (4/5, 80%) used apps with functionalities including self-monitoring as well as reminders and alerts with either automatic feedback [33,37] or educational information [29,31], while 1 RCT used the most comprehensive combination of strategies including self-monitoring, reminders and/or alerts, automatic feedback and educational information [42]. Two other studies (2/14, 14%) [28,39] using apps with the same comprehensive combination of functionalities represented a high risk of bias and reported no statistically significant effects of using the app.

**Table 3:** Blood pressure effects and quality of randomized controlled trial (RCT).

RCT study			Follow up point, month	N	Systolic blood pressure		Diastolic blood pressure	
					Change	Effect	Change	Effect
**Logan et al [33], mean (SD)**								
	**Intervention**							
		Over 24 hours	12	55	–8.7 (14.7)	Positive^a^	–4.2 (9.3)	Positive
		During the daytime	12	55	–9.1 (15.6)	Positive^a^	–4.6 (9.2)	Positive
	**Control**							
		Over 24 hours	12	55	–1.7 (12.1)	—	–1.1 (6.8)	—
		During the daytime	12	55	–1.5 (12.2)	—	–1.3 (6.6)	—
**Or and Tao [42], mean (95% CI)**								
	Intervention		3	33	–16.7 (–22.8 to –10.7)	Positive	–8.0 (–11.5 to –4.5)	Neutral^b^
	Control		3	30	–2.1 (–8.6 to 4.4)	—	–2.1 (–8.6 to 4.4)^c^	—
**Mendelson et al [35]**								
	Intervention		4	54	NR^d^	Neutral	NR	Neutral
	Control		4	53	NR	—	NR	—
**Davidson et al [24], mean**								
	Intervention		6	33	–34.8	Positive	–12	Positive
	Control		6	30	–9.7	—	–4.5	—
**McGillicuddy et al [25] (mm Hg), mean (SE)**								
	Intervention		12	9	132.2 (3.7)	Positive	NR	NR
	Control		12	9	154.2 (5.7)	—		—
**McGillicuddy et al [27] (mm Hg), mean**								
	Intervention		3	9	121.80	Positive	80.70	Neutral
	Control		3	10	138.78	—	79.44	—
**Moore et al [30], mean (SD)**								
	Intervention		3	20	–26.3 (11.9)	Positive	–13.7 (9.4)	Neutral
	Control		3	22	–16.0 (12.1)	—	–8.2 (8.6)	—
**Petrella et al [34]**								
	Intervention		13	75	NR	Neutral	NR	Neutral
	Control		13	74	NR	—	NR	—
**Bloss et al [28], mean**								
	Intervention		6	65	NR	Neutral	–3.6	Neutral
	Control		6	65	NR	—	–6.1	—

^a^The app had significant positive effect on blood pressure.

^b^The app had neutral effect on blood pressure.

^c^*P*<.001.

^d^NR: not reported.

**Table 4:** Blood pressure effects and quality of nonrandomized studies.

Randomized controlled trial study		Follow up point, month	N	Systolic Blood Pressure		Diastolic Blood Pressure	
				Change	Effect	Change	Effect
**Bengtsson et al [37], mean (SD)**							
	Intervention	2	50	–7 (18)	Positive^a^	–4.9 (10)	Positive
	Control	2	50	NR^b^	—	NR	—
**Patel et al [29] (mm Hg), mean**							
	Intervention	7	50	135	Positive	85	Positive
	Control	7	30	NR	—	NR	—
**Mao et al [31], mean (SE)**							
	Intervention	4	763	–5.96 (1.64)	Positive	NR	NR
	Control	4	73	NR	—	NR	—
**Albini et al [41], mean (SD)**							
	Intervention	6	303	–10.5 (6.3)	Positive	–6.2 (3.8)	Positive
	Control	6	298	–6.1 (6.9)	—	–3.4 (4.5)	—
**Anglada-Martínez et al [39] (mm Hg), mean (SD)**							
	Intervention	6	42	131.3 (9.8)	Neutral^c^	75.4 (6.7)	Neutral
	Control	6	42	130.2 (13.9)	—	79.9 (9.6)	—

^a^The app had significantly positive effect on blood pressure.

^b^NR: not reported.

^c^The app had neutral effect on blood pressure.

The evidence is therefore inconclusive about which of these functionality combinations would be more effective in lowering BP, but it suggests that apps incorporating more comprehensive functionalities are likely to be effective.

### Usability, Satisfaction, and Attitudes

Two of the 21 studies (10%) explored the usability of the apps [36,40] and 9 (43%) assessed user satisfaction with and attitudes toward the apps [26,27,30-32,38,39,43,44], 1 of which (1/9, 11%) also evaluate usability among experts [44]. All of these 11 studies focused on the patient perspective, whereas 5 of them (46%) also considered the HCPs’ perspective [30,36,39,40,44].

Generally, the use of the app was highly accepted by participants in all 9 studies that assessed user satisfaction [26,27,30-32,38,39,43,44]. User satisfaction was measured through the participants rating their experience with the app [30,31], administration of satisfaction questionnaires [26,27,32,39,44], or conducting interviews [38,43]. The satisfaction rate ranged from 7.2 to 9.8 [30,31,39] for studies using a 10-point satisfaction rating scale, and from 3.1 to 4.8 [27,44] for studies utilizing a 5-point satisfaction rating scale.

The participants reported that the apps were easy to use [27,32,38,39,43], convenient [38,43], helpful in effectively communicating with HCPs [26,27] and in hypertension management [27,38,43,44], including medication adherence and adjustment [26,27,43], and helped increase their active role in care, health awareness, and motivation [30,38,43]. Although some participants felt that the apps were useful only for patients with an unstable BP [38,43], elderly patients, patients with polypharmacy or caregivers [39], most patients and HCPs stated that they would continue using the app after the study [30,39,43] and would recommend it to their friends [39]. In 3 of the 9 studies (33%) [30,38,43] the participants suggested that the app would be more useful if improvements could be made. These improvements include tailoring the graph according to the participants’ preference, for example, coloring graphs, sending motivational messages according to the inputted data [38]. It also was suggested to support the self-monitoring of other conditions such as blood glucose [43], include alerts that inform patients if the BP readings are abnormal, and improving the performance of the app by loading faster [30].

One study (1/3, 33%) evaluated the app through conducting heuristic evaluation among technology and health informatics experts, and 2 studies (2/3, 67%) only conducted a usability test of the app amongst users. In the heuristic evaluation, some usability problems were identified. Of the 2 studies that assessed usability among users, 1 used direct observation of the participants [40] and the other used an observation method with specific questions asked to the participants [36]. All the participants found both apps easy to use [36,40].

Six studies assessing usability and satisfaction (6/11, 55%) were of poor quality and only 5 of the 11 (46%) presented a low-moderate risk of bias. Generally, the participants seemed satisfied with the apps, they accepted using them in managing their condition and found them easy to use.

## Discussion

### Principal Findings

The aim of this systematic review was to synthesize evidence about the effectiveness, acceptance, and usability of using mobile and tablet apps to reduce BP.

This review found studies about 16 apps with similar functionalities. However, they were different in the number of combined functionalities. The majority of the apps used different combinations of functionalities, whereas 1 app had only 1 function [43]. In all 9 studies that assessed users’ satisfaction, the participants generally seemed to accept using apps to support the self-management of their BP. It also indicates that using the apps seems to be effective in supporting the self-management of hypertension and has the potential to lower BP as this was reported in 10 studies (6 RCTs and 4 nonrandomized studies). It should be noted that, of these, only 2 RCTs (33%) and 3 nonrandomized studies (75%) were of good quality. Due to the variety of study designs and quality the results, there is inconclusive evidence about which of these functionality combinations would be more effective in lowering BP. However, it would appear that apps incorporating more comprehensive functionalities are likely to be effective.

This study found that using apps may help reduce SBP and DBP significantly. Notably, this result was in accordance with other studies using mobile and other similar older technologies [11,46]. In 1 meta-analysis, a decrease of 5 mm Hg in DBP or 10 mm Hg in SBP was found to reduce coronary heart disease events by 22% and stroke by 41% [47], as a decrease of 1 mm Hg in SBP leads to a 5% reduction in the risk of stroke [46]. The findings of this review are in line with other systematic reviews that involved mobile phone and tablet-based intervention in managing chronic diseases, which showed that the use of apps has the potential to improve health outcomes among those living with chronic diseases [8,10,11,48,49].

The results with regard to acceptance are supported by studies assessing the acceptance and usability of mobile apps in the management of chronic diseases [49,50]. A study assessing the usability of a commercially available app for diabetes found a lack of usability for its main target users of elderly diabetics [51]. This finding, thereby, highlights the importance of assessing the usability of apps for hypertension and close cooperation and intensive usability tests with the targeted users during the development process of the apps.

In some studies, the apps were used in combination with other platforms, such as a website. The reported effects, therefore, cannot be solely attributed to the apps. The use of apps with automatic feedback without the involvement of clinicians to monitor patients remotely may be effective in controlling BP. Similarly, apps in which HCPs were involved in monitoring patients remotely and providing their feedback or instructions, with either automatic feedback or not, could also have a significant impact on BP. In short, it is possible that both approaches are effective.

The results of this review should be interpreted with caution, as some studies with a high risk of bias (6/9, 67% of RCTs; 6/10, 60% of nonrandomized studies) were included, and methodological issues have been identified in most of the included studies. These issues emerged from potential biases in some RCT studies because of the failure to implement the blinding of subjects and the assessor, lack of concealment and randomization procedures, small sample size, and short study duration. However, the blinding of subjects was impossible across the interventions due to the nature of using apps. Nonrandomized quantitative studies also had limitations, such as their small sample size, short duration, and attrition bias [39]. Many of the studies included in this paper were conducted in different health and social care settings, which means that comparisons between them are not straightforward. Consequently, the generalizability of the results of some of these studies is limited. Although evidence of the effectiveness of mHealth is increasing, there is a lack of evidence concerning the sustainability of the findings after the app intervention has ceased. This suggests that further research is warranted to determine long-term benefits and eliminate these limitations.

### Strengths and Limitations of this Review

This review has some limitations that should be considered when interpreting the results. First, studies published in languages other than English were not included, which increases the likelihood of relevant research being missed. Moreover, all types of studies were included regardless of their quality as it is often helpful to have more recent findings. However, low-quality studies present more inconclusive data, which affects the results. It was not possible to conduct a meta-analysis due to the study designs heterogeneity; combining results that have been obtained from different types of randomized and nonrandomized studies will not yield useful data. In addition, the inclusion of controlled and non-controlled studies might yield a combination of possibly inconclusive results. Their inclusion may offer a wider body of evidence. Despite these limitations, this study is the first systematic review exploring the effectiveness of using mobile apps in the self-management of hypertension and their acceptance among users. Consequently, it might be a useful roadmap to guide further studies on the use of mobile apps by people with hypertension. The authors developed a comprehensive search strategy and then hand searched the reference lists of each identified full-text articles and systematic review to find potentially relevant studies for inclusion in this systemic review and considered combinations of functionalities that were used in the apps.

### Recommendations for Further Study

The methodological quality of studies included in this review was generally low. This indicates that future studies should consider some essential criteria, including a sufficient number of participants and duration time, concealment and randomization procedures, blinding of the assessor, and low attrition rates. Future studies assessing the effectiveness of apps should focus on apps that incorporate more comprehensive functionalities, that are identified in this review as the most promising functionalities for self-management of hypertension, including self-monitoring, reminders and alerts with either automatic feedback or educational information or both. It is important also to assess and understand users’ satisfaction with and acceptance of these apps. A well-designed RCT with multiple arms using apps with different combinations of functionalities to enable identification of the most effective combinations would also be beneficial.

### Conclusion

This systematic review indicates that the use of apps to support the self-management of hypertension are accepted by patients and could assist in lowering and controlling their BP. It would appear that apps incorporating more comprehensive functionalities are likely to be effective. The results should be interpreted with caution, as most of the studies were of high risk of bias. More research is required to identify the effectiveness of using apps in lowering BP and to understand what functionality combinations are effective for lowering BP.

## Appendix

Multimedia Appendix 1 Study characteristics.

Multimedia Appendix 2 MEDLINE search strategy.

Multimedia Appendix 3 Study outcomes.

Multimedia Appendix 4 Cochrane checklist too for RCTs.

Multimedia Appendix 5 NIH tools for non-randomized studies.

Multimedia Appendix 6 NIH tools for non-randomized studies (observational cohort and cross-sectional studied).

Multimedia Appendix 7 NIH tools for non-randomized studies (Pre-post studies) .

Multimedia Appendix 8 CASP tool for qualitative studies.

## Abbreviations

BP: blood pressure
CASP: Critical Appraisal Skills Programme
DBP: diastolic blood pressure
HCP: health care professional
mHealth: mobile health
SBP: systolic blood pressure
RCT: randomized controlled trial
