# Supplementary material for: Mobile Apps to Support the Self-Management of Hypertension: Systematic Review of Effectiveness, Usability, and User Satisfaction
Source: JMIR Mhealth Uhealth. 2018 Jul 23;6(7):e10723. doi: 10.2196/10723 (PMC6079301; doi:10.2196/10723)
Supplement: Multimedia Appendix 1 [file mhealth_v6i7e10723_app1.pdf]

Study and country	Study Design	Study setting	Study duration	Subjects	Interventions	Study device (Operating system)	Study Quality
**Anglada-Martínez et al Spain [39]**	Single-arm prospective pre-post intervention study	Casanova Primary Care Clinic and Hospital Clinic	6 months	**Patients**:<br>**N=** 42 (F 21.4%, M 78,6%)<br>**Age:** (mean= 56)<br>**SBP**[a]**:** NR[b] **DBP**[c]**:** NR<br><br>**HCP**: **N**= 5	**I**[d]**=** Medplan platform (website for HCPs + smartphone app)<br>**C**[e]**=** usual care	iPhone (iOS) or NR (Android)	Poor
**Mao et al USA [31]**	RCT[f] (Retrospective analysis)	Data from a pilot Commercial collaboration	12 months	**Patients:**<br>**N=** 1012 (F 68%, M 32%)<br>**I=** 763, C= 73<br>**Age** (mean= 44.63; I= 44.87 C= 42.36)<br>**SBP:** I= 131.27 (1.52) **C=**NR<br><br>**HCP**: **N**=NA	**I=** Vida app + a Bluetooth-connected pedometer and wire§1less scale + a Bluetooth-enabled blood pressure cuff<br>**C=** usual care	iPhone (iOS) or NR (Android)	Fair
**Kang et al., South Korea [44]**	Technology development study (survey)	Cardiovascular clinics at tertiary hospitals	4 weeks	**Patients**:<br>**N=**38 (F% 34, M 66%)<br>**Age:** 56 years<br><br>**HCP**: **N**=CD	**I=**Smartphone app<br>+ BP monitor<br>**C**=NA[g]	NR (NR)	Poor
**Banerjee et al USA [32]**	Technology development studies (Survey)	A diabetes and hypertension clinic.	NA	**Patients:**<br>**N=**385<br>**Age:** NA<br>**SBP:** NR **DBP:** NR<br><br>**HCP**: **N**=NA	**I=**Smartphone app (My vital signs)<br>**C**=NA	iPhone (iOS) or NR (Android)	Poor
**McGillicuddy et al (2013) USA [26]**	Survey	Kidney Transplant Clinic at the Medical University of South Carolina (MUSC)	3 months	**Patients:**<br>**N=** 99 (F 35%, M 65% (64/98))<br>**Age:** (mean= 44.63)<br>**SBP:** NR **DBP:** NR<br><br>**HCP**: **N**=NA	**I=** SMASH (cellular connected electronic medication device + a wireless (bluetooth-enabled) BP monitor + smartphone (app + messaging)<br>**C**=NA	Motorola Droid X (Android)	Fair
**Sun et al China [43]**	Longitudinal quasi-experiment design	Tsinghua Elderly University & a nearby community	6 weeks	**Patients:**<br>**N=**19 (F=57.8%, M= 42.1%)<br>**Age:** 49-70 years (mean=59.2)<br><br>**HCP**: **N**=NA	**I=** Mobile app +<br>BP monitor<br>**C=** NA	NR (NR)	Poor

Study and country	Study Design	Study setting	Study duration	Subjects	Interventions	Study device (Operating system)	Study Quality
**Hallberg et al South Sweden [38]**	Qualitative study (semi-structured interview)	4 different primary health-care centers	NR	**Patients:** **N=** 49 (F=23, M=26) **Age:** 30-70 years (mean= 58) HCP: N=NA	Mobile app + Website + BP monitor	NR (NR)	Good
**Bengtosson et al South Sweden [36]**	Qualitative study (interview)	Primary health care center in a multi-cultural city suburb and one internal medical outpatient clinic at a provincial hospital in a smaller town	NR	**Patients**: **N**=21 (F=42.86%, M=57.14%) **Age:** 49-82 **HCP**: **N**=4	Mobile app	NR (NR)	Good
**Logan et al Canada [33]**	RCT	The offices or clinics of physicians practicing in metropolitan Toronto.	12 months	**Patients**: **N=** 110 (F 44.5%, M 55.4%), **I=** 55 C= 55 **Age: I=**62.7 ±7.8 **C=**63.1±9.0 24-h **SBP:** **I=**139.4±11.6 , C= 139.4±10.6 24-h **DBP: I=** 73.9±10.6, C=75.4± 8.7 **HCP**: **N**=NA	**I=**smartphone (app)+ Bluetooth-enabled BP monitor + voice message. **C=** usual care	BlackBerry (NR)	Good
**Mendelson et al France [35]**	RCT	Sleep clinics	4 months	**Patients**: **N=**107 (F 16.8%, M 83.2%) I=54, **C=**53 **Age:** 63 ± 9 Office **SBP:** mmH = 135±13.2I= 135.3 ±14.1 **C=**138.4 ±6.4 office **DBP:** mmH=81.5 ±9.6 I= 80.9 ± 9.7, **C=** 82 ±9.6 Morning **SBP:** mmHg **I=**	**I=** Telemedicine (Smartphone app + automated BP monitor + CPAP fitted with a nasal mask and given an autotitrating machine. **C=**usual care	**NR(NR)**	Fair

Study and country	Study Design	Study setting	Study duration	Subjects	Interventions	Study device (Operating system)	Study Quality
				135.3±12.3 (SE)<br>**C=**135.3 14.1 (SE)<br>Evening **SBP:** mmHg=**I=** 130.8±13.2(SE) **C=** 132.0 17.1 (SE)<br>Morning **DBP: I=** 80.9 mm Hg 9.7(SE) **C=** 82.0±9.0(SE)<br>Evening **DBP: I=** 75.9 mm Hg 8.7(SE) **C=** 79.0 mmHg 10.2 (SE)<br>Type2 diabetes 36.4%<br><br>**HCP**: **N**=NA			
**Bloss et al USA [28]**	RCT	Scripps Translational Science Institute	6 months	**Patients:**<br>**N=** 160 (F 70%, M 30%)<br>**Age:** 33-81 years (Mean=56(9.0) (SD)<br>Hypertension 67(89)<br>non-insulin dependent diabetes (NIDDM) (9)<br>arrhythmia (10)<br>insulin-dependent diabetes (IDDM) (5),<br>Comorbidity (21)<br><br>**HCP**: **N**=NA	**I=** BP monitor +iPhone with corresponding apps+ HealthyCircles account (online and mobile)+<br>**C=** usual care+ education	iPhone 4 or iphone 4s (iOS)	Poor
**McGillicuddy et al USA [25]**	RCT (Retrospective analysis)	Kidney Transplant Clinic at the Medical University of South Carolina (MUSC)	12 months	**Patients:**<br>**N=** 18 (F 44.4%, M 55.6%)<br>**Age:** 33-81 years<br>Mean=**I=** 42.44(12.04) ,**C=**57.89 (8.72) (SD)<br><br>**HCP**: **N**=NA	**I=**cellular connected electronic medication device + BP monitor+ smartphone app+ email + SMS or call	Motorola Droid X (Android)	Poor

Study and country	Study Design	Study setting	Study duration	Subjects	Interventions	Study device (Operating system)	Study Quality
**Carrera et al Spain [40]**	Technology development studies	NA	NA	**N=** 20	**I=**BP monitor + smartphone and tablet (app + messaging) **C=**NA	Samsung Galaxy S2, Samsung Galaxy S3 and Nexus 5 (Android v. 5.1.1), Ipad 2 (iOS) and Iphone 6 (iOS v. 8.4)	Poor
**McGillicuddy et al USA [27]**	A proof-of-concept RCT	Kidney Transplant Clinic at the Medical University of South Carolina (MUSC)	3 months	**Patients:** **N=** 19 (F 68%, M 32%) **I=** 9, C= 10 **Age:** (Mean= **I=** 42.44 **C=** 57.6) **SBP: I=** 138.35, **C=**135.11 **DBP:** I=87.55, **C=**76.11<br><br>**HCP**: **N**=NA	**I=** cellular connected electronic medication device + BP monitor+ smartphone app + email + SMS or call **C=**usual care	Motorola Droid X (Android)	Poor
**Moore et al USA [30]**	RCT	The Ambulatory Practice of the Future (APF) at the Massachusetts General Hospital is designed	12 weeks	**Patients:** **N=** 42 (F 40.5%, M 59.5%) **I=** 20 C= 22 Age: NA (Mean=50.0 ±12.8) **SBP:** 147.6; I= 145.7, **C=**149.8 **DBP:** 87.2; **I=**86.0, **C=**88.43<br><br>**HCP**: **N**=NA	**I=** Tablet app (CollaboRhythm) a wireless BP monitor **C=** usual care	NR (NR)	Poor
**Petrella et al Canada [34]**	Parallel-group, RCT	Gateway Rural Health Research Institute, Seaforth (community-based research centre)	52 weeks	**Patients:** **N=**149 (F 74.5%; M 25.5%) **I=** 75, C= 74 **Age:** Mean **I=** 55.7, **C=** 57.8<br><br>**HCP**: **N**=NA	**I=** Smartphone app(healthyanywhere) + Bluetooth™ enabled BP monitor and a glucometer+ a pedometer +Aerobic exercise prescription **C=** exercise prescription and logged exercise in a journal	Blackberry Curve 8300 or 8530 (NR)	Poor

**Study and country**	**Study Design**	**Study setting**	**Study duration**	**Subjects**	**Interventions**	**Study device (Operating system)**	**Study Quality**
**Davidson et al USA [24]**	RCT	NR	6 months	**Patients:**<br>**N=** 38 (F 68%, M 32%)<br>**I=** 33, **C=** 30<br>**Age:** NA<br>Mean<br>**I=**47.50±11.08,<br>**C=**48.45±11.32<br>**SBP: I=, C=** NA<br>**DBP: I=, C=** NA<br><br>**HCP**: **N**=NA	**I=** cellular connected electronic medication device + BP monitor+ smartphone app + email + SMS or call<br>**C=** usual care	Motorola Droid X (Android)	Poor
**Or & Tao [42]**	RCT	Patients homes	3 months	**Patients:**<br>**N=** 63 (F5%, M95%)<br>**I=** 33, **C=** 30<br>**Age:** 30-70 years (Mean=69.5)<br>**SBP/DBP:**<br>**I=** 134.9/79.5,<br>**C=**130.1/76.1<br><br>**HCP**: **N**=NA	**I=** tablet +<br>a 2-in-1 BG and BP monitor<br>**C=** a 2-in-1 BG and BP monitor + a log book	NR (NR)	Fair
**Albini et al Italy [41]**	Pilot study	General Practitioners clinics	9 months	**Patients:**<br>**N =** 601 (F :38.5% M: 61.5%)<br>**I =** 303, C = 298<br>Age I= 57.9±11.3<br>**C=** 56.9±11.5<br>**SBP: I=** 148.3 ±11.7,<br>**C=**145.4±3.9<br>**DBP:**<br>**I =** 91.6 ± 6.5, **C =** 88.8 ± 6.1<br>**DBP:**<br>IG = 91.6 ± 6.5, CG = 88.8 ± 6.1<br><br>**HCP: N**=9	**I=** Eruhypertension APP(E-APP)+ Misuriamo platform<br>+ BP monitoring<br><br>**C=** usual care	NR(NR)	Poor
**Patel et al USA [29]**	Open label trial	Internal medicine, renal/hypertension, and cardiology clinics of the George Washington	10 months	**Patients:**<br>**N=** 50 (M: 31%, F:69%)<br>**Age:** 53 years (33 -78)<br>**SBP:** 144, **DBP:** 89<br><br>**HCP**: **N**=NA	**I=**Smartphone app<br>**C=**NA	NR (NR)	Fair

Study and country	Study Design	Study setting	Study duration	Subjects	Interventions	Study device (Operating system)	Study Quality
		University Medical Faculty					
**Bengtsson et al Sweden [37]**	Explorative, longitudinal study	4 different primary health-care centers	8 weeks	**Patients:**<br>**N=** 50 (F 48%, M 52%)<br>**Age:** 33-81 years (Mean=59.5)<br>**SBP:** 142, **DBP:** 84<br><br>**HCP**: **N**=NA	**I=** mobile phone-based system for the self-report questions<br>+<br>BP monitor<br>+<br>web-based system<br>**C=**NA	NR (NR)	Good

[a]SBP: systaltic blood pressure; [b]NR: not reported; [c]DBP: diastolic blood pressure; [d]I intervention group/phase [e]C: control group/phase; [f]RCT: randomised control trial; [g]NA: not applicable; [h] HCP: health care professionals.
