# Supplementary material for: Mobile Apps to Support the Self-Management of Hypertension: Systematic Review of Effectiveness, Usability, and User Satisfaction
Source: JMIR Mhealth Uhealth. 2018 Jul 23;6(7):e10723. doi: 10.2196/10723 (PMC6079301; doi:10.2196/10723)
Supplement: Multimedia Appendix 2 [file mhealth_v6i7e10723_app2.pdf]

1. exp Hypertension/
2. hyperten*.kw,ti.
3. high blood pressure.ab,ti.
4. HTN.ab,ti.
5. 1 or 2 or 3 or 4
6. exp Mobile Applications/
7. mobile health.ab,ti.
8. *Medical Informatics Applications/
9. (ehealth or e-health or e?health).ab,ti.
10. (mhealth or m-health or m?health).ab,ti.
11. (window? adj3 phone?).ab,ti.
12. (window? adj3 mobile?).ab,ti.
13. apps.ab,ti.
14. (iphone? or i-phone?).ab,ti.
15. (ipad? or i-pad?).ab,ti.
16. (ipod?touch or i-pod? or ipod?).ab,ti.
17. nokia.ab,ti.
18. palm OS.ab,ti.
19. blackberry*.ab,ti.
20. symbian.ab,ti.
21. personal digital assistant?.ab,ti.
22. PDA.ab,ti.
23. (smartphone? or smart?phone?).ab,ti.
24. exp cellular phone/
25. cell phone?.ab,ti.
26. mobile phone?.ab,ti.
27. applet?.ab,ti.
28. (software adj3 app*).ab,ti.
29. (mobile adj3 software).ab,ti.
30. (mobile adj3 app*).ab,ti.
31. (smartphone? or smart?phone?).ab,ti.
32. (telemonitor* or tele?monitor*).ab,ti.
33. (telemedicine or tele?medicine).ab,ti.
34. (telehealth or tele?health).ab,ti.
35. exp Computers, Handheld/
36. or/6-35
37. (remote adj2 (monitor* or treat* or care)).ab,ti.
38. exp self care/
39. self care.ab,ti.
40. self monitor*.ab,ti.
41. self manage*.ab,ti.
42. self treat*.ab,ti.
43. self medication.ab,ti.
44. self administration.ab,ti.
