# Supplementary material for: Mobile Apps to Support the Self-Management of Hypertension: Systematic Review of Effectiveness, Usability, and User Satisfaction
Source: JMIR Mhealth Uhealth. 2018 Jul 23;6(7):e10723. doi: 10.2196/10723 (PMC6079301; doi:10.2196/10723)
Supplement: Multimedia Appendix 3 [file mhealth_v6i7e10723_app3.pdf]

Study	Follow-up	Outcomes				
		**SBP** (mm Hg)	**Effect**	**DBP** (mm Hg)	**Effect**	**Satisfaction/ usability**
**Anglada-Martínez et al [39]**	6 m[a]	**I**[b]=131.3(9.8) (SD[c]) **C**[d]=130.2 (13.9) (SD)	=[h]	**I**=75.4, (6.7) (SD) **C**=79.9, (9.6) (SD)	=	**Satisfaction and attitudes:** Patients and HCPs were satisfied with the system (mean 7.2±2.7 and 7.3±1.7 respectively), its ease to use and its useful functions, but some patient had problem in receiving reminders and HCP found adherence recorded were inconsistent. They wanted to keep using and recommend it for others.
**Mao et al [31]**	4 m	125.31 (1.18) (SE[e]); -5.96[f] (1.64) (SE) ***P***=.002	+[i]	NR[j]	NR	**Satisfaction and attitudes:** Almost half of participants were rated the app, ranging between 9.77 to 9.81 where 10 being most satisfied.
**Kang et al [44]**	NA[k]	NA	NA	NA	NA	**Satisfaction and attitudes:** Patient were satisfied with the app with mean ranging from 3.1 to 4.2l and perceived its usefulness; a score mean of 3.7 out of 5.
**Banerjeeet al [32]**	NA	NA	NA	NA	NA	**Satisfaction and attitudes:** more than 65% agreed that the app was easy to us
**McGillicuddy et al [26]**	NR	NR	NR	NR	NR	**Satisfaction and attitudes:** Most participants (78/99) had positive attitudes toward system, it helpfulness in reminding them to follow their doctor direction (87% (86/99), its adequate privacy (79/99), its effectiveness in facilitating communication with healthcare providers 87% (86/99) and allowing their doctor to adjust their medication (84%, 83/99).
**Sun et al [43]**	2 w[g] 4 w 6 w	NA	NA	NA	NA	**Satisfaction and attitudes:** Satisfaction vastly improved throughout the study. Participants felt the app was easy to use, convenient, helpful in increasing awareness and supporting health management and treatment requesting to using app after study, some suggestion were provided: sending alarm if BP is abnormal and support self-monitor of BG and electrocardiogram

**Hallberg et al [38]**	NA	NA	NA	NA	NA	**Satisfaction and attitudes:** Patients considered system appropriate, easy to use and useful in increasing their participation, health awareness and motivation. However, some provided suggestion including: more reminders, tailoring messages and graphs based on personal answers and preference.
**Bengtosson et al [36]**	NA	NA	NA	NA	NA	**Usability:** Almost all participants reported app easy to use.
**Carrera et al [40]**	NA	NA	NA	NA	NA	**Usability**: The first 10 participants reported the system is use-friendly. The usability rating was on average ranging between 3 to 4.5 out of 5.
**McGillicuddy et al [27]**	1 m	**I**=129.70, **C**= 147.22	+	**I**=83.10, **C**=81.72	=	NA
	2 m	**I**=129.55, **C**= 138.22	+	**I**=84.50, **C**=77.49	=	NA
	3 m	**I**=121.80, **C**= 138.78	+	**I**=80.70, **C**=79.44	=	**Satisfaction and attitudes:** The participants reported high overall satisfaction with the system with average 4.8/5 and easy to learn to use (4.7/5) and easy to use (4.8/5), useful for medication and health management (4.3/5).
**Moore et al [30]**	12 w	**I**=-26.3[f] (11.9) **C**=-16.0[f] (12.1) ***P***= .009	+	**I**=-13.7[f] (9.4) (SD) **C**=8.2[f], (8.6) (SD) ***P***=.054	=	**Satisfaction and attitudes:** Intervention group had higher satisfaction rate 8.9 vs 7.6 out of 10. Most participants were satisfied with app usefulness in increasing their active role in the care and their health awareness and they wanted to keep using it.
**Petrella et al [34]**	12 w	**I**=-2.96[f] (-7.48 to 1.57), **C**=-8.73[f] (-13.6 to-3.91)	**-**	**I**= 84.0(10.7), **C**= 80.9 (9.0)	**-**[m]	NA
	24 w	NR ***P***<.001	=	NR	=	NA
	52 w	NR ***P***<.001	=	NR ***P***< .001	=	NA

**Davidson et al [24]**	1 m	NR<br>***P***<.001	+	NR<br>***P***< .001	**NR**	NA
	3 m	NR<br>***P***<.001	+	NR<br>***P***< .001	**NR**	NA
	6 m	**I**= -34.8[f],<br>**C**=-9.7<br>***P***<.001	+	**I**=-12,<br>**C**=-4.5<br>***P***<.001	+	NA
**Or & Tao [42]**	1 m	**I**=118.2 (113.2 to 123.2); -16.7[f] (95% CI[n],-22.8 to -10.7); 118<br>**C**= 127.9 (122.6 to 133.3); -2.1(95% CI,-8.6 to 4.4)	+	**I**=71.5 (68.0 to 75.0); -8.0[f] (95% CI,-11.5 to -4.5);<br>**C**= 75.8 (72.1 to 79.6); -2.1[f] (95% CI,-8.6 to 4.4)<br>***P***<.001	+	NA
	2 m	**I**= 124.6 (119.6 to 129.5); -10.3[f]; 95% CI, (−16.4 to −4.3)<br>**C**=123.8 (118.4 to 129.2); -6.2[f] (-12.8 to 0.3)	=	**I**=72.9 (69.4 to 76.4); -6.6[f] (5% CI,-10.1 to -3.1)<br>**C**=74.2 (70.4 to 78.0); -1.9[f] (5% CI,-5.7 to 1.9)<br>***P***=0.028	+	NA
	3 m	**I**=121.9 (116.9 to 126.9); -13.0[f]; (95% CI, -19.1to -6.9),<br>**C**=124.6 (119.3 to 130.0); -5.4[f] 95% CI, -12.0 to 1.1)<br>***P***<.034	+	**I**=73.8 (70.2 to 77.3); -5.7[f] (5% CI, -9.3 to -2.2) **C**= 74.2 (70.4 to 78.0); -2.0[f] (5% CI, -5.7 to 1.8)	=	NA
**Logan et al [33]**	12 m	**24h BP**<br>**I**=-8.7 ±14.7<br>**C**=-1.7±12.1<br>***P***+.005	+	**I**=-4.2±9.3<br>**C**=-1.1± 6.8<br>***P*** =.006	+	NA
		**Daytime BP**<br>**I**=-9.1±15.6<br>**C**=-1.5±12.2<br>***P*** =.003	+	**I**=-4.6±-9.2<br>**C**=-1.3 ±-6.6<br>**P** =.003	+	NA

**Mendelson et al [35]**	4 m	NR	=	NR	=	NA
**Bloss et al [28]**	6 m	NR<br>***P*=.**56	=	**I**=-3.6,<br>**C**=-6.1<br>***P*** = .35	=	NA
**McGillicuddy et al [25]**	12 m	**I**= 132.2, 3.7 (SE),<br>**C**= 154.2 mm Hg 5.7 (SE)<br>***P*=** .01	+	NR	NR	NA
**Albini et al [41]**	6 m	**I**=137.8(12.8) (SD); -10.5[f] ± 6.3<br>**C**= 139.3 (12.3) (SD); -6.1[f] ± 6.9 (SD)<br>***P***<.0001	+	**I**=85.4 ±7.5;-6.2[f] ±3.8; **C**= 85.4 ±7.9; -3.4[f] ± 4.5<br>***P***<.0001	+	NA
**Patel et al [29]**	Pre-app 1m	137<br>***P***=.032	+	85<br>***P***=032	+	NA
	App use 3m	136<br>***P***=.004	+	84<br>***P***=.004	+	NA
	App off 3m	135<br>***P***=.006	+	85<br>***P***=.006	+	NA
**Bengtsson et al [37]**	8 w	**-**7(18) (SD)	+	-4.9[f](10) (SD)	+	NA

[a]m:month; [b]I: intervention group/phase; [c]SD: standard deviation; [d]C: control group/phase; [e]SE=standard error; [f] mean change of SBP or DBP level; [g]w: week; [h]=: the app had neutral effect on BP; [i]+: the app had significant positive effect on BP; [m]-: the app negative effect on BP; [j]NR: not reported; ; [k]NA: not applicable; [n]CI: Confidence interval.
