# Supplementary material for: Mobile Apps to Support the Self-Management of Hypertension: Systematic Review of Effectiveness, Usability, and User Satisfaction
Source: JMIR Mhealth Uhealth. 2018 Jul 23;6(7):e10723. doi: 10.2196/10723 (PMC6079301; doi:10.2196/10723)
Supplement: Multimedia Appendix 4 [file mhealth_v6i7e10723_app4.pdf]

**Study**	**Selection bias**		**Performance bias**	**Detection bias**	**Attrition bias**	**Reporting bias**	**Other bias**
	**Random sequence generation**	**Allocation concealment**	**Blinding of participants and personnel**	**Blinding of outcome assessors to intervention allocation**	**incomplete outcome data**	**Selective reporting of the outcome, subgroups, or analysis**	**Others**
McGillicuddy et al (2013) [27]	**?**	**?**	**?**	√	√	√	x
	Random sequence generation was not noted within the study	Allocation concealment was not noted within the study	The blinding was not described.	The nurses or coordinators blinded to the subject's cohort assignment	Titration rate was good 88.7%.	All reported outcomes in methodology reported in result section.	The study was funded and ethically approved and has similar baseline characteristic. However small sample size may affect detecting the impacts of technology and generalize the findings.
McGillicuddy et al (2015) [25]	**?**	**?**	**?**	√	√	√	x
	Random sequence generation was not noted within the study	Allocation concealment was not noted within the study	The blinding was not described.	The nurses or coordinators blinded to the subject's cohort assignment t	Titration rate was good 88.7%.	All reported outcomes in methodology reported in result section.	The study was funded and ethically approved and has similar baseline characteristic. However small sample size may affect detecting the impacts of technology and generalize the findings.
Davidson et al<br><br>[24]	?	x	?	?	√	√	x
	.<br>Random sequence generation was not noted within the study	Allocation concealment was not noted within the study	The blinding was not described.	The blinding was not described.	only one person drop out<br>for the intervention group	All pre-specified outcomes are done	The study was funded and ethically approved and has some similar baseline characteristic; it is not similar at DBP, age. However, very small sample size; affects detecting the impacts of

							technology and generalize the findings.
Bloss et al [28]	x	x	?	x	x	√	√
	Random sequence genera on was not noted within the study	Treatment allocation with unblinded research coordinator.	The blinding was not described.	Unblended research coordinator.	Number of missing in control is more than intervention (23% intervention vs. 13% in control); reasons for loss to follow-up were not described, and did not intention to treat analysis used	All outcomes in the methods section were reported in the results section.	The study was funded and ethically approved, good sample size; and similar baseline characteristic.
Or & Tao [42]	√	√	**x**	**x**	√	√	x
	Using permuted blocks of size 4 and 6 (with sequentially numbered, opaque, sealed envelopes)	Using permuted blocks of size 4 and 6 (with sequentially num- bered, opaque, sealed envelopes) presented in random order	Given the nature of the study and its intervention, the patients and outcome assessors were not blinded.	Given the nature of the study and its intervention, the patients and outcome assessors were not blinded	only one person drop out for each group	All reported outcomes in methodology reported in result section,	The study was funded and ethically approved, but small sample size may affect detecting the impacts of technology and generalize the findings.
Logan et al [33]	√	√	**?**	**?**	√	√	√
	Randomized; locks of 4 and 6 patients randomly arranged and administered by a person not directly involved in the study.	Randomized; locks of 4 and 6 patients randomly arranged and administered by a person not directly involved in the study.	a prospective, randomized, open, blinded primary end-point trial; which means no patients were blinded.	a prospective, randomized, open, blinded primary end-point trial; which means no physician were blinded.	Only one person drop out for the intervention group	all pre-specified outcomes are done	The study was funded and ethically approved, good sample size; and similar baseline characteristic.
Petrella et al	√	x	?	x	?	√	√

[34]	Block randomization (based on appointment time).	Due to the randomization procedure, research staff could not be blinded to group allocation.	Due to this randomization procedure, research staff could not be blinded to group allocation.	Due to this randomization procedure, research staff could not be blinded to group allocation.	more drop out in the control (14 vs. 8 in the intervention). Did not use intention to treat analysis.	all outcomes in the methods section were reported in the results section.	The study was funded and ethically approved, good sample size; and similar baseline characteristic.
Moor et al [30]	√	?	?	?	√	√	x
	Each subject was assigned the next sequential study number, which was pre-randomized to either the intervention or the control group.	Allocation concealment was not noted within the study	The blinding was not described.	The blinding was not described.	only one person drop out for each group	Based on paper only, protocol not obtained. All pre-specified outcomes were reported in the results	The study was funded and ethically approved, small sample size may affects detecting the impacts of technology and generalize the findings
Mendelson et al [35]	√	√	?	?	x	√	√
	Randomization was stratified by the recruiting center in blocks of 6 participants.	Treatment allocation prepared by an individual otherwise unaffiliated with the study	The blinding was not described.	The blinding was not described.	Almost the same number missing in both groups, however, more people withdrew from the intervention (8 vs 1 from the usual care.	all outcomes in the methods section were reported in the results section.	The study was funded and ethically approved, good sample size; and similar baseline characteristic

[a]√: low risk of bias, [b]**x**: High risk of bias, [c]**?**: unclear risk of bias
