# Supplementary material for: Mobile Apps to Support the Self-Management of Hypertension: Systematic Review of Effectiveness, Usability, and User Satisfaction
Source: JMIR Mhealth Uhealth. 2018 Jul 23;6(7):e10723. doi: 10.2196/10723 (PMC6079301; doi:10.2196/10723)
Supplement: Multimedia Appendix 5 [file mhealth_v6i7e10723_app5.pdf]

**Criteria/ Study**	**Albini et al [41]**
1-Was the study described as randomized, a randomized trial, a randomized clinical trial, or an RCT?	x[b]
2. Was the method of randomization adequate (i.e., use of randomly generated assignment)?	x
3. Was the treatment allocation concealed (so that assignments could not be predicted)?	x
4. Were study participants and providers blinded to treatment group assignment?	x
5. Were the people assessing the outcomes blinded to the participants' group assignments?	x
6. Were the groups similar at baseline on important characteristics that could affect outcomes (e.g., demographics, risk factors, co-morbid conditions)?	CD[e]
7. Was the overall drop-out rate from the study at endpoint 20% or lower of the number allocated to treatment?	√[a]
8. Was the differential drop-out rate (between treatment groups) at endpoint 15 percentage points or lower?	NR
9. Was there high adherence to the intervention protocols for each treatment group?	NA[d]
10. Were other interventions avoided or similar in the groups (e.g., similar background treatments)?	NA
11. Were outcomes assessed using valid and reliable measures, implemented consistently across all study participants?	√
12. Did the authors report that the sample size was sufficiently large to be able to detect a difference in the main outcome between groups with at least 80% power?	NR[c]
13. Were outcomes reported or subgroups analyzed prespecified (i.e., identified before analyses were conducted)?	√

14. Were all randomized participants analyzed in the group to which they were originally assigned, i.e., did they use an intention-to-treat analysis?	x
**Quality rating & additional comment**	Poor quality due to lack of randomization and blinding procedures (selection bias) that affect validity

[a]**√:** Yes; [b]**x:** No; [c]**NR,** not reported; [d]**NA,** not applicable; [e]**CD,** cannot determine
