# Supplementary material for: Mobile Apps to Support the Self-Management of Hypertension: Systematic Review of Effectiveness, Usability, and User Satisfaction
Source: JMIR Mhealth Uhealth. 2018 Jul 23;6(7):e10723. doi: 10.2196/10723 (PMC6079301; doi:10.2196/10723)
Supplement: Multimedia Appendix 6 [file mhealth_v6i7e10723_app6.pdf]

**Criteria/ Study**	Bengtsson et al [37]	Carrera et al [40]	McGillicuddy et al [26]	Mao et al [31]	Kang et al [44]	Sun et al [43]	Banerjee et al [32]
1. Was the research question or objective in this paper clearly stated?	√[a]	x[b]	√	√	√	√	x
2. Was the study population clearly specified and defined?	√	x	√	√	√	NR	x
3. Was the participation rate of eligible persons at least 50%?	√	CD[e]	CD	√	CD	CD	CD
4. Were all the subjects selected or recruited from the same or similar populations (including the same time period)? Were inclusion and exclusion criteria for being in the study pre-specified and applied uniformly to all participants?	√	CD	√	√	CD	CD	CD
5. Was a sample size justification, power description, or variance and effect estimates provided?	√	NR[c]	x	√	√	x	NR
6. For the analysis in this paper, were the exposure(s) of interest measured prior to the outcome(s) being measured?	NA	NA[d]	NA	NA	√	NA	√
7. Was the timeframe sufficient so that one could reasonably expect to see an association between exposure and outcome if it existed?	x	CD	CD	√	x	√	CD
8. For exposures that can vary in amount or level, did the study examine different levels of the exposure as related to the outcome (e.g., categories of exposure, or exposure measured as continuous variable)?	NA	NA	NA	NA	NA	NA	NA

9. Were the exposure measures (independent variables) clearly defined, valid, reliable, and implemented consistently across all study participants?	√	√	√	√	√	√	√
10. Was the exposure(s) assessed more than once over time?	NA	NA	NA	NA	NA	NA	NA
11. Were the outcome measures (dependent variables) clearly defined, valid, reliable, and implemented consistently across all study participants?	√	x	√	x	√	√	x
12. Were the outcome assessors blinded to the exposure status of participants?	NA	x	x	NA	NR	NR	x
13. Was loss to follow-up after baseline 20% or less?	√	NA	NA	√	x	√	NA
14. Were key potential confounding variables measured and adjusted statistically for their impact on the relationship between exposure(s) and outcome(s)?	√	x	NR	√	x	x	x
**Quality rating & additional comment**	**Good quality** despite it had short duration follow-up.	**Poor quality** because it had the potential of selection, measurement and attrition bias as well as many missing information	**Fair quality**	**Fair quality** but it had the potential risk for measurement bias.	**Poor quality** due to the potential of selection and attrition bias and short duration follow-up, as well as many missing information.	**Poor quality** because it has the potential of selection and short duration follow-up as well as some missing information.	**Poor quality** because it had the potential of selection, measurement and attrition bias.

[a]**√:** Yes; [b]**x:** No; [c]**NR,** not reported; [d]**NA,** not applicable; [e]**CD,** cannot determine
