# Supplementary material for: Mobile Apps to Support the Self-Management of Hypertension: Systematic Review of Effectiveness, Usability, and User Satisfaction
Source: JMIR Mhealth Uhealth. 2018 Jul 23;6(7):e10723. doi: 10.2196/10723 (PMC6079301; doi:10.2196/10723)
Supplement: Multimedia Appendix 7 [file mhealth_v6i7e10723_app7.pdf]

**Criteria/ Study**	**Anglada-Martínez et al [39]**	**Patel et al [29]**
1. Was the study question or objective clearly stated?	√[a]	√
2. Were eligibility/selection criteria for the study population prespecified and clearly described?	√	√
3. Were the participants in the study representative of those who would be eligible for the test/service/intervention in the general or clinical population of interest?	**CD**	√
4. Were all eligible participants that met the prespecified entry criteria enrolled?	**x**[b]	√
5. Was the sample size sufficiently large to provide confidence in the findings?	**x**	√
6. Was the test/service/intervention clearly described and delivered consistently across the study population?	√	√
7. Were the outcome measures prespecified, clearly defined, valid, reliable, and assessed consistently across all study participants?	CD[e]	CD
8. Were the people assessing the outcomes blinded to the participants' exposures/interventions?	NR[c]	CD
9. Was the loss to follow-up after baseline 20% or less? Were those lost to follow-up accounted for in the analysis?	x	√
10. Did the statistical methods examine changes in outcome measures from before to after the intervention? Were statistical tests done that provided p values for the pre-to-post changes?	√	√
11. Were outcome measures of interest taken multiple times before the intervention and multiple times after the intervention (i.e., did they	x	x

use an interrupted time-series design)?		
12. If the intervention was conducted at a group level (e.g., a whole hospital, a community, etc.) did the statistical analysis take into account the use of individual-level data to determine effects at the group level?	[d]NA	NA
**Quality rating & additional comment**	**Poor quality** because of small number of participants and potential of selection and titration bias as well as some missing information that affect study validity	**Fair quality**; Despite of relatively small sample size and single group, it has good attitration rate and use interrupted time series design as well as well reported

[a]**√:** Yes; [b]**x:** No; [c]**NR,** not reported; [d]**NA,** not applicable; [e]**CD,** cannot determine
