# Supplementary material for: Mobile Apps to Support the Self-Management of Hypertension: Systematic Review of Effectiveness, Usability, and User Satisfaction
Source: JMIR Mhealth Uhealth. 2018 Jul 23;6(7):e10723. doi: 10.2196/10723 (PMC6079301; doi:10.2196/10723)
Supplement: Multimedia Appendix 8 [file mhealth_v6i7e10723_app8.pdf]

**Criteria/ Study**	**Hallberg et al [38]**	**Bengtsson et al [36]**
1-Was there a clear statement of the aims of the research?	√[a]	√
2. Is a qualitative methodology appropriate?	√	√
3-Was the research design appropriate to address the aims of the research?	√	√
4- Was the recruitment strategy appropriate to the aims of the research?	√	√
5. Was the data collected in a way that addressed the research issue?	x[b]	x
6. Has the relationship between researcher and participants been adequately considered?	x	x
7. Have ethical issues been taken into consideration?	√	√
8. Was the data analysis sufficiently rigorous?	√	√
9. Is there a clear statement of findings?	√	√
10. How valuable is the research?	x	√

[a]√: Yes; [b]x:No; [c]CT, Cannot tell
